# Supplementary material for: Characterization of the Genetic Variability within Ziziphus nummularia Genotypes by Phenotypic Traits and SSR Markers with Special Reference to Geographic Distribution
Source: Genes (Basel). 2023 Jan 6;14(1):155. doi: 10.3390/genes14010155 (PMC9858891; doi:10.3390/genes14010155)
Supplement: Supplementary file 1 [file genes-14-00155-s001.zip › genes-2080452-supplementary.pdf]

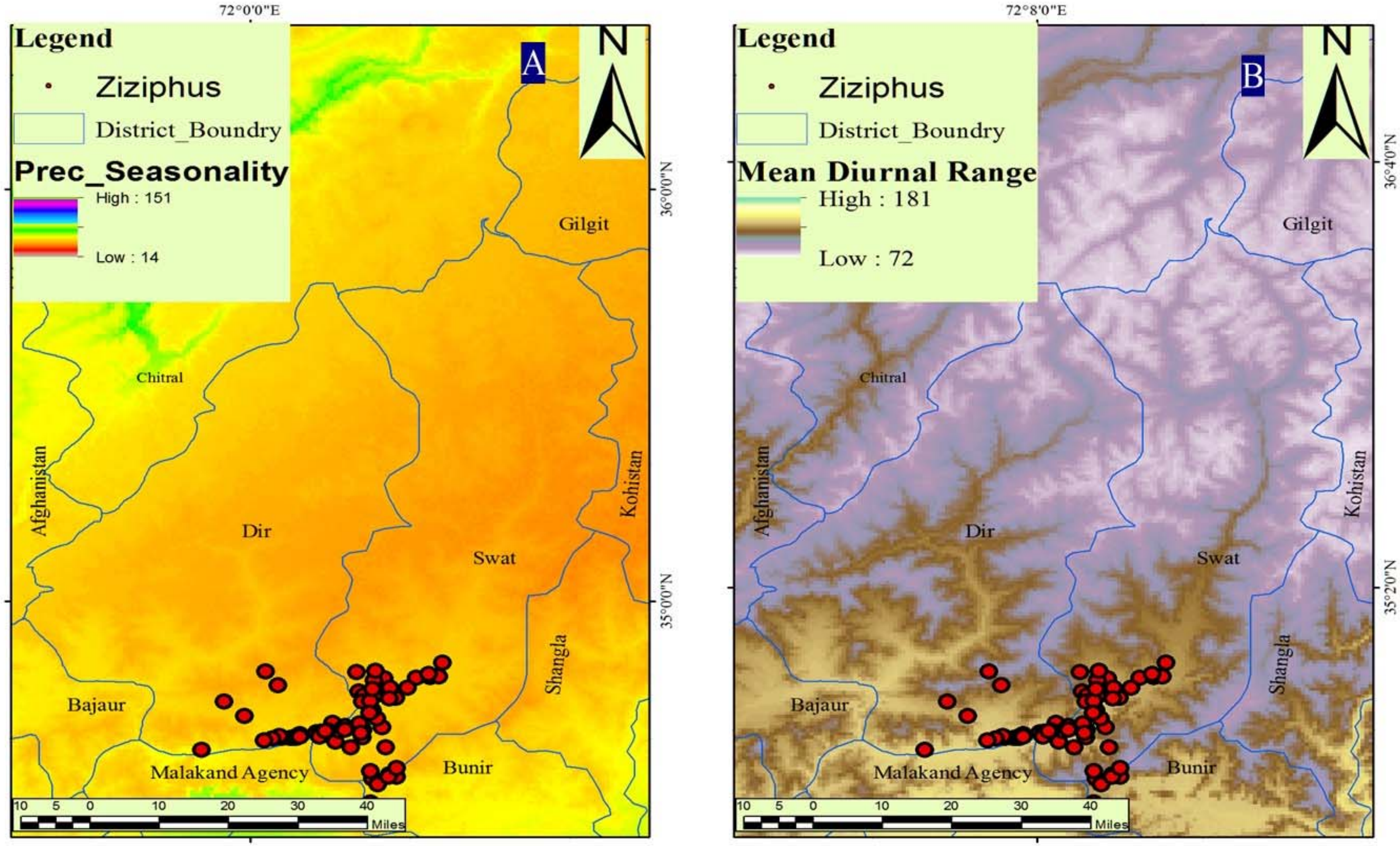

Figure S1. Showed that the ArcGIS mapping of a bioclimatic variable on the selected regions, showed variation in (A), Prec-Seasonality and (B), Mean diurnal range, respectively

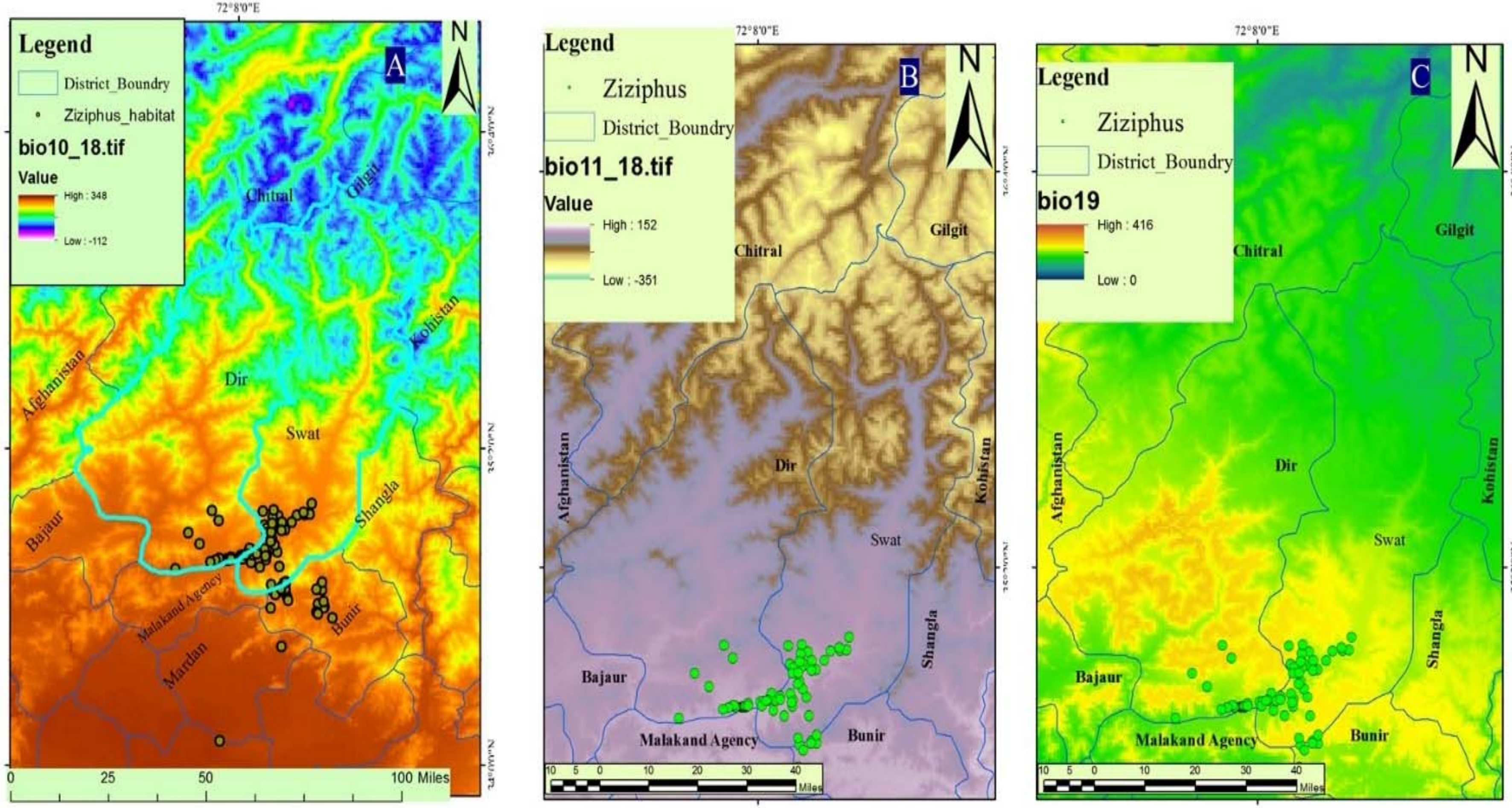

Figure S2. Applied ArcGIS mapping of a bioclimatic variable on the selected regions, (A), distribution in the selected remote regions of *Z. nummularia* genotypes, and (B and C), showing accrued temperature ranges.
